# Summary of biological research on hepatoblastoma: a scoping review

**DOI:** 10.3389/fped.2024.1309693

**Published:** 2024-02-08

**Authors:** Huan-sheng Wang, Jing Lao, Ren-sen Jiang, Bin Wang, Xiao-peng Ma, Jian-yao Wang

**Affiliations:** ^1^ Department of General Surgery, Shenzhen Children’s Hospital of China Medical University, Shenzhen, Guangdong Province, China; ^2^ Department of General Surgery, Shenzhen Children’s Hospital of ShanTou University, Shenzhen, Guangdong Province, China; ^3^ Department of General Surgery, Shenzhen Children’s Hospital, Shenzhen, Guangdong Province, China

**Keywords:** hepatoblastoma (HB), genomics, β-catenin, transcriptomics, epigenetic

## Abstract

**Background:**

Hepatoblastoma is the most prevalent primary hepatic malignancy in children, comprising 80% of pediatric hepatic malignancies and 1% of all pediatric malignancies. However, traditional treatments have proven inadequate in effectively curing hepatoblastoma, leading to a poor prognosis.

**Methods:**

A literature search was conducted on multiple electronic databases (PubMed and Google Scholar). A total of 86 articles were eligible for inclusion in this review.

**Result:**

This review aims to consolidate recent developments in hepatoblastoma research, focusing on the latest advances in cancer-associated genomics, epigenetic studies, transcriptional programs and molecular subtypes. We also discuss the current treatment approaches and forthcoming strategies to address cancer-associated biological challenges.

**Conclusion:**

To provide a comprehensive summary of the molecular mechanisms associated with hepatoblastoma occurrence, this review highlights three key aspects: genomics, epigenetics, and transcriptomics. Our review aims to facilitate the exploration of novel molecular mechanisms and the development of innovative clinical treatment strategies for hepatoblastoma.

## 1 Introduction

Hepatoblastoma (HB) is a predominant malignant liver cancer in children, with a heightened incidence in those within the first three years of life. While the combination of cisplatin-based chemotherapy and surgical resection, a substantial of affected children still confront the challenges of metastasis, grapples with highly aggressive tumor marked by multiple nodules at diagnosis, vascular invasion, chemoresistance, and recurrence (1). High-risk cases, distinguished by adverse prognostic factors such as tumor expansion, the presence of metastasis, extreme levels of tumor markers, and older age, continue to present formidable therapeutic hurdles and grim prognoses. To develop new therapeutic avenues for high-risk hepatoblastoma, it is essential to explore novel treatment strategies grounded in a comprehensive understanding of the underlying biological mechanisms (2). Capitalizing on new-generation gene sequencing technologies, including Target-seq, CN analysis, DNA methylation identification technologies and Whole Genome Sequencing (WGS), researchers have constructed an extensive molecular map of hepatoblastoma. This endeavor has unveiled the genetic heterogeneity of the disease and identified potential therapeutic targets (3). Some of these breakthroughs have already translated into tangible clinical benefits for cancer patients. In this comprehensive review, we delved into the latest biological research on hepatoblastoma, with a particular emphasis on cancer-associated genomics, epigenetic studies and transcriptional programs. These facets encompass gene mutations, chromosomal alterations, epigenetic methylation and various RNA types. Additionally, we explored current treatment strategies and addressed unresolved cancer-associated biological issues, with the overarching goal of advancing both the research and clinical progress in the field of hepatoblastoma.

### 1.1 Article types

Our scoping review follows the Preferred Reporting Items for Systematic Reviews and Meta-Analyses (PRISMA) guidelines (4).

### 1.2 Eligibility criteria

**Figure 1:**
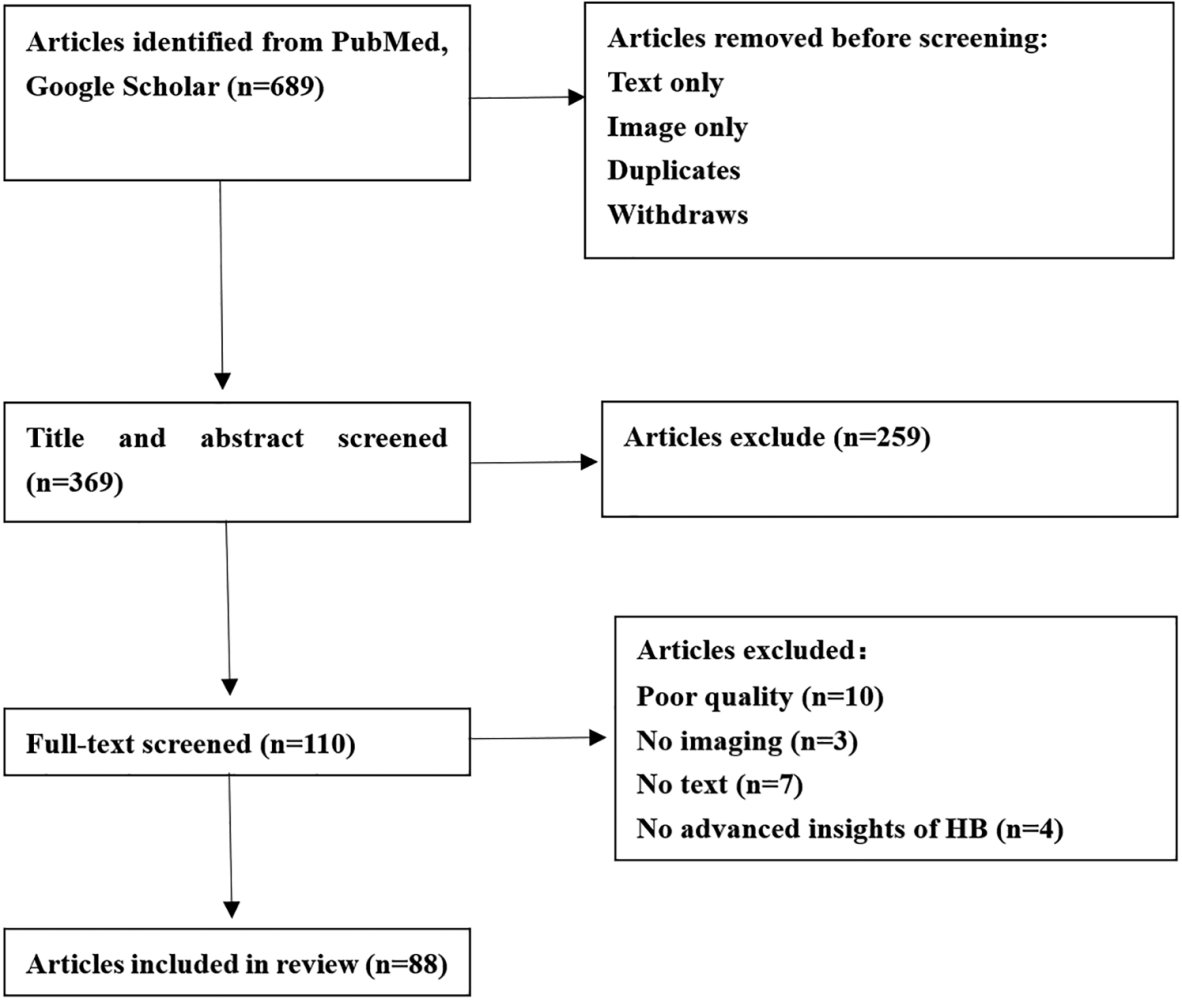
Flowchart of article selection.

Our scoping review focused on the latest advances in cancer-associated genomics, epigenetic studies, transcriptional programs and Molecular subtypes. The inclusion criteria for our review consisted of English language articles published between 1999 and 2023, including both conference papers and journal articles. We chose this time frame to capture the most up-to-date research in this rapidly evolving field and the Flowchart of article selection was depicted in Figure 1.

### 1.3 Information sources

A search of multiple databases was carried out, including PubMed and Google Scholar. The most recent search was executed on December, 2023.

### 1.4 Search strategy

All the studies collected in this research were confined to the medical field. Initially, our search comprised three keyword groups: hepatoblastoma, hepatoblastoma and genomics study, hepatoblastoma and epigenetic study, hepatoblastoma and transcriptomics studies. We combined these keywords to carry out the collection across two databases.

### 1.5 Study selection

Two reviewers (HSW and JL) conducted the title and abstract screening independently. Studies were subjected to full-text review in cases of disagreement, and a consensus was reached through discussions. Subsequently, each article was reviewed and labeled according to the tasks. These tasks encompassed report generation, visual question answering and other related tasks, with the possibility for a single article to correspond to multiple tasks. During the screening and the full-text review stages, we excluded non-medical articles and poor-quality articles. HSW and JYW participated in the revision of the article.

### 1.6 Data extraction and synthesis

**Figure 2:**
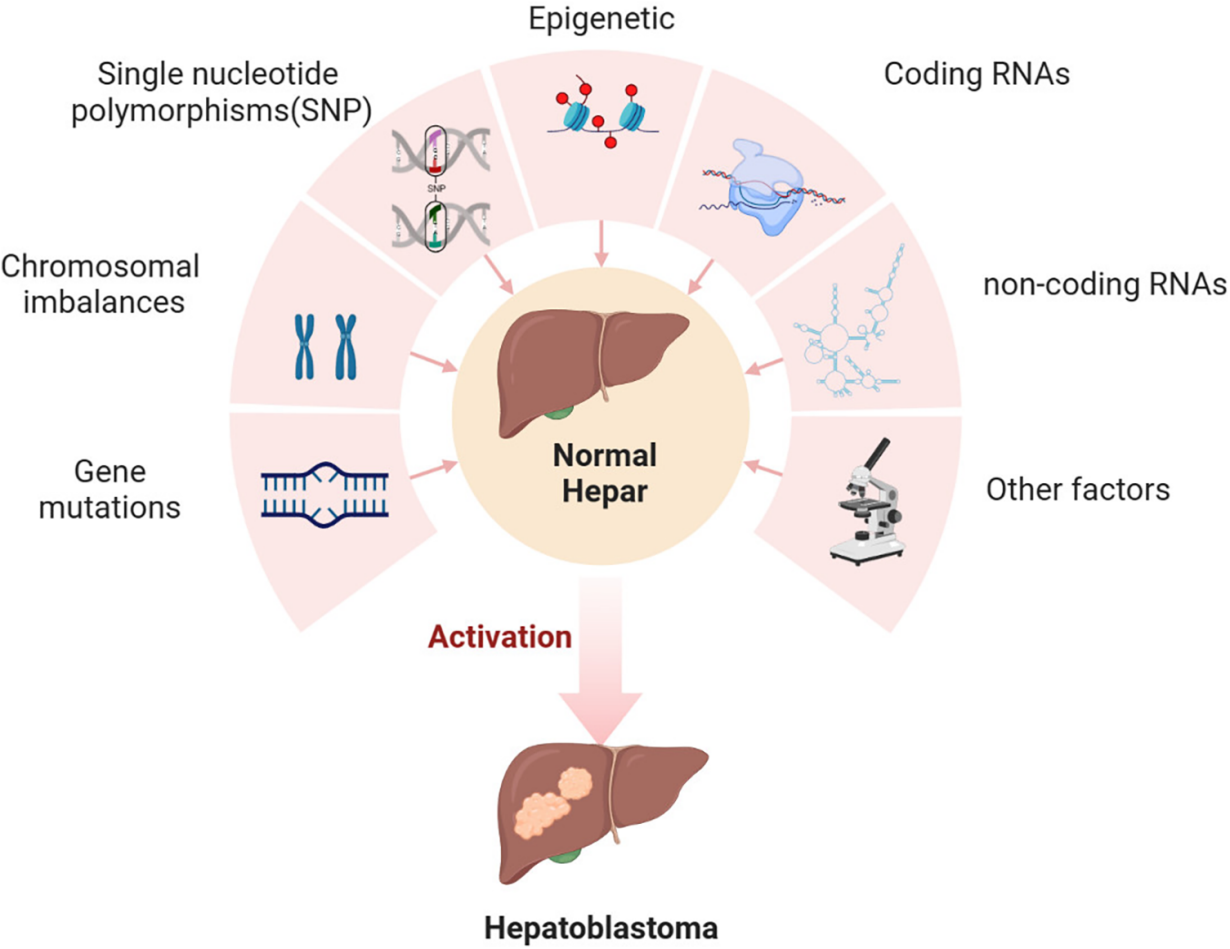
The molecular mechanism of HB occurrence.

We created a form to extract the relevant data from the eligible articles. Characteristics of the articles included author and year, title, objectives of the study, the study design, what factors the study compares, and the main outcome of the study. We believe this review will facilitate the exploration of novel molecular mechanisms and the development of innovative clinical treatment strategies for hepatoblastoma. The current primary research focus on the molecular biology mechanisms of HB was illustrated in Figure 2.

## 2 Result

### 2.1 Genomics study of HB

In the past, genomics has significantly reshaped our understanding of cancer biology, by enabling comprehensive analyses of various genetic aspects in large-scale studies across different cancer types. These analyses encompass mutations, copy number alterations, single nucleotide polymorphisms, gene expression and DNA methylation profiles. In 1999, Koch et al. conducted an analysis involving 52 hepatoblastoma (HB) biopsies and three HB cell lines from sporadic HB cases. Sporadic HB is particularly notable for its high frequency of in-frame mutations in the CTNNB1 gene, which encodes β-Catenin, a central component of the canonical Wnt pathway (5). Subsequently, genomic studies on hepatoblastoma tumor specimens have unveiled a wealth of information. Exome sequencing of primary HB not only demonstrated an unexpectedly low mutation rate, with an average of only 2.9 mutations per tumor (6), but also confirmed that CTNNB1 mutations and deletions were present in 78 out of 88 (89%) of the tumors examined (7). Additionally, mutations in the NFE2L2 gene, which has been linked to resistance against cisplatin, were identified in 5% of hepatoblastomas (8). Furthermore, mutations in the TERT gene, specifically in the promoter region, were detected in several older patients (9). Beyond these, a range of other gene mutations in HB were identified, including ARID1A, ITPR2, APC, TP53, CX3CL1, A2ML1, CEP164 and EP300 (10–12). Moreover, comprehensive genomic profiling revealed alterations in genes such as ERBB4, MDM4, FBXW7, SRC, and BRCA2 due to mutations, amplification, or loss. These genes represent potential therapeutics that may hold clinical significance for certain HB patients (13, 14). Another noteworthy finding is the alteration of the 11p15.5 locus, which houses the IGF2 gene, observed in a significant proportion of hepatoblastomas. These alterations can occur through mechanisms such as copy-neutral loss of heterozygosity or changes in methylation status, involving either loss or gain (15, 16). Table 1 provided a summary of the mutated genes presented in this article.

**Table 1:** Summary of gene mutations.

Gene mutations	Pathway/Protein	Ref
CTNNB1 (89%)	Wnt/β-catenin	(5)
NFE2L2 (5%)	Hippo/YAP	(8)
ARID1A	PEDF induced signaling	(10)
ITPR2	Inositol triphosphate receptor	(10)
APC	Wnt signaling	(10)
TP53	ERK signaling	(10)
BRCA2	BRCA1	(13)
ERBB4	ERK signaling	(13)
MDM4	MDM4	(14)
FBXW7	Notch signaling	(13)
SRC	SRC	(13)
IGF2	IGF1R/IGF2 signaling	(16)
CX3CL1	CX3CL1/CX3CR1 chemokine signaling	(11)
A2ML1	A2ML1	(11)
CEP164	DNA IR-damage and cellular response via ATR	(11)
EP300	Wnt signaling	(12)

A diverse range of chromosomal imbalances has been identified in hepatoblastomas, including gains in various chromosomes such as 1q, 2/2q, 8/8q,12, 20, 22q, 6p, 7q, and 17 as well as losses in 1p, 4q, 11q and 20 (6–8, 10, 15, 17–19). The correlation of these chromosomal abnormalities with patient outcomes has revealed specific associations. Notably, gains in 8q and 20 have been linked to a poorer prognosis, while losses in 1p and 18 tend to indicate increased hepatoblastoma aggressiveness (18, 19). Additionally, loss of heterozygosity (LOH) in 11p15.5 has been correlated with a higher frequency of relapse (18). Among the entire group, six hot-spot chromosome regions have been frequently affected: 1q31.3q42.3, 2q23.3q37.3, and 20p13p11.1 show gains, while a 5.3 Mb amplification occurs in 2q24.2q24.3, and losses are observed in 1p36.33p35.1, 4p14 and 4q21.22q25 (19). Further investigation is needed to assess the prognostic of these chromosomal imbalances in hepatoblastomas.

In recent years, single nucleotide polymorphisms (SNPs) associated with hepatoblastoma have been discovered. These include protective factors H19 rs217727 G>A, YTHDF1 rs6090311 A>G, WTAP rs7766006 G>T and *XPC* rs1870134 as well as risk factors likes MPO G-463-A G>A, LINC00673 rs11655237 C>T, HMGA2 rs968697 T>C, H19 rs2839698 G>A, H19 rs302427° C>G, LIN28B rs314276 T>G, LIN28B rs314276 C>A, hOGG1 rs293795 A>G and *XPC* rs2607775 C>G (20–28). The correlation of these SNPs with patient outcomes has shown that CCND1 rs9344 G>A is associated with a poorer prognosis (29). There are likely many more gene polymorphisms related to hepatoblastomas waiting to be discovered and studied. Table 2 provided a summary of identified chromosomal imbalance and SNPs associated with HB.

**Table 2:** Summary of chromosomal imbalance and single nucleotide polymorphisms.

Chromosomal imbalance										
Gains	1q	2/2q	8/8q	12	20	22q	6p	7q	17	18
Losses	1p	4q	11q	20						
Single nucleotide polymorphisms										
Risk factors	MPO G-463-A G>A		LINC00673 rs11655237 C>T		HMGA2 rs968697 T>C		H19 rs2839698 G>A		H19 rs302427° C>G	
	LIN28B rs314276 T>G		LIN28B rs314276 C>A		HOGG1 rs293795 A>G		*XPC* rs2607775 C>G		CCND1 rs9344 G>A	
Protective factors	H19 rs217727 G>A		YTHDF1 rs6090311 A>G		WTAP rs7766006 G>T		*XPC* rs1870134			

The field of cancer genomics has witnessed significant advancements, particularly with the application of single-gene sequencing, thanks to biotechnological developments. However, there exists a current challenge in effectively integrating these previously discovered results into clinical prognosis analysis. The future imperative lies in merging molecular information with CHIC risk stratification. This integration necessitates the deployment of technological tools that facilitate more efficient and cost-effective sequencing, coupled with robust computational algorithms capable of extracting biologically relevant insights from vast datasets. These components are foundational to the successful establishment of tumor risk stratification and treatment schedules. Furthermore, as we move forward, we anticipate the emergence of additional treatment methods based on these aforementioned findings.

### 2.2 Epigenetic study of HB

Exome sequencing of primary hepatoblastoma (HB) has unveiled a remarkable finding: an average of merely 2.9 mutations per tumor, indicating an unexpectedly low mutation rate (6). This observation challenges the notion that HBs solely arise from the accumulation of genetic mutations. Since most HB cases occur in children, this prompts us to consider the significant role played by epigenetic mechanisms in controlling their development and progression. Epigenetic studies typically encompass heritable changes resulting from non-genetic sequence alterations. These alterations encompass a range of processes, including DNA methylation, histone modifications, acetylation, phosphorylation, ubiquitylation and sumoylation. These epigenetic modifications have been associated with the occurrence and progression of various prevalent diseases, including cancer (30).

#### 2.2.1 DNA methylation

Gene expression is intricately regulated at an epigenetic level through DNA methylation. Within the realm of known WNT antagonists, the secreted frizzled-related protein 1 (SFRP1) frequently experiences downregulation across various cancer types, implying its tumor-suppressive functions (31). While the precise roles of DNA methylation in oncogenesis remain incompletely understood. In a controlled trial, literature reports indicate heightened expression of specific genes associated with DNA methylation (DNMT3A, DNMT1, TET1, TET2, TET3) in HB. This heightened expression leads to the accumulation of 5-hydroxymethylcytosine (5hmC). This observation suggests a potential link between the perturbation of DNA methylation and HB (32, 33). It is evident that promoter hypermethylation is linked to inappropriate transcriptional repression of tumor suppressor genes. Conversely, hypomethylation of repetitive sequences can lead to the activation of retro-transposon elements and subsequent genomic instability (34). Notably, studies have suggested that hypermethylation of the SFRP1 promoter region contributes to transcriptional silencing in hepatoblastoma tumor cell lines. This silencing, in turn, promotes tumor cell proliferation, colony formation, and enhanced migratory potential (35). Another study indicated heightened methylation within the NNMT's promoter region in HB, which downregulated NNMT expression and potentially contributed to tumor development (36). Moreover, hypermethylation has been reported in the promoter regions of other genes including RASSF1A (37, 38), CASP8 (37), SOCS1 (39–41), APC, CDH1, MT1G (41), HHIP (6), CDKN2A (42) and IGF2 (37, 41), while hypomethylation of IGFBP3 (43) has been observed in a subset of tumors. CpG islands, often found in close proximity to gene transcriptional regulatory regions, are linked to approximately 56% of human genome coding genes. Consequently, investigating the methylation status of CpG islands in gene transcriptional areas holds significant importance. One study conducted an extensive methylation analysis across the entire genome of HB cell lines and identified 38 genes associated with HB tumors. Among these genes, 14 displayed hypermethylated CpGs, 23 exhibited hypomethylation, and one gene, TSPAN9, showed both hypermethylation in its gene body and hypomethylation in its promoter region. Additionally, this study demonstrated that CpG sites on chromosomes 6, 9, 11, 20 and 22 were more methylated in hepatoblastomas compared to non-tumoral livers (44).

Given the substantial influence of DNA methylation on the molecular behavior of HB, investigating these epigenetic profiles may offer valuable insights for prognostication and risk stratification. Building on the concept of “DNA hypomethylation and 14q32 locus overexpression”, Carrillo-Reixach et al. have proposed the potential utility of a molecular risk stratification system, which could complement existing clinical models (45).

#### 2.2.2 RNA methylation

N^6^-methyladenosine (m^6^A) is an RNA modification that plays a significant role in normal cell metabolism and the development of neoplasia (46). Recent research has revealed an increase in m^6^A modifications in hepatoblastoma, with METTL3 being the primary factor responsible for these abnormal m^6^A modifications. Furthermore, this research has identified CTNNB1 as a regulator of METTL3-guided m^6^A modification in HB (47). Another study has suggested a potential correlation between METTL14 polymorphisms and hepatoblastoma susceptibility, offering a fresh perspective on the genetic factors underlying m^6^A modification in hepatoblastoma (48).

#### 2.2.3 Protein modification

Post-translational modification (PTM) of proteins represents a crucial mechanism for regulating protein function and increasing the proteome's complexity within organisms (49). One such PTM, O-GlcN Acylation, is a broad, dynamic and reversible process that plays a pivotal role in regulating various cellular processes, including protein-protein interactions, cell signaling, epigenetic reprogramming, and metabolic regulation. Notably, O-GlcN Acylated proteins have been implicated in the progression and development of hepatoblastoma. A study has highlighted that the O-GlcNAcylation modification of HSPB1 promotes proliferation and enhances the chemotherapeutic resistance of hepatoblastoma cell lines *in vitro* experiments (49). Histones are essential proteins responsible for maintaining chromatin structure and contributing to the dynamic, long-term regulation of genes. Each of the four core histones, H2A, H2B, H3, and H4, forms the core of the nucleosome, with the *N*-terminal tail of histone 3 (H3) being subject to methylation, acetylation, phosphorylation, and arginine residue modification. Employing chromatin immunoprecipitations (CHIP), one study has suggested that E3 ubiquitin-like containing PHD and RING finger domain 1 (UHRF1) may play a crucial role in the profound silencing of tumor suppressor genes (TSGs) such as HHIP, IGFBP3, and SFRP1 in hepatoblastoma. This silencing is achieved through a combination of epigenetic mechanisms, including repressive histone modification and DNA methylation. Furthermore, this study has identified that upon UHRF1 depletion, five out of the 16-genes studies were downregulated (50, 51). Another study indicated that the tumor suppressor protein CCAAT/Enhancer Binding Protein α (C/EBPα) loses its inhibitory effect on tumor development post-dephosphorylation by protein phosphatase 2A (PP2A). The resultant dephosphorylated C/EBPα, upon interaction with histone deacetylases 1(HDAC1), forms a complex that suppresses the expression of PEPCK and CYP3A4 genes, key for generating normal liver cell biomarkers. Additionally, HDAC1 binding to SP5, a transcription factor, hampers the expression of the tumor suppressor protein P21, potentially promoting tumor development (52). However, the relationship between hepatoblastoma and other histone modifications, such as histone ubiquitination, acylation or ADP-ribosylation, remains unclear, representing a relatively unexplored area in this field. In recent years, studies have explored the degradation of the tumor suppressor proteins (TSPs) by the oncoprotein gankyrin (Gank). This process has been associated with the advancement of liver tumor development. Literature reports suggest that Gank facilitates the degradation of CUGBP1, a tumor suppressor protein, potentially contributing to the onset of mice hepatoblastoma (53).

**Figure 3:**
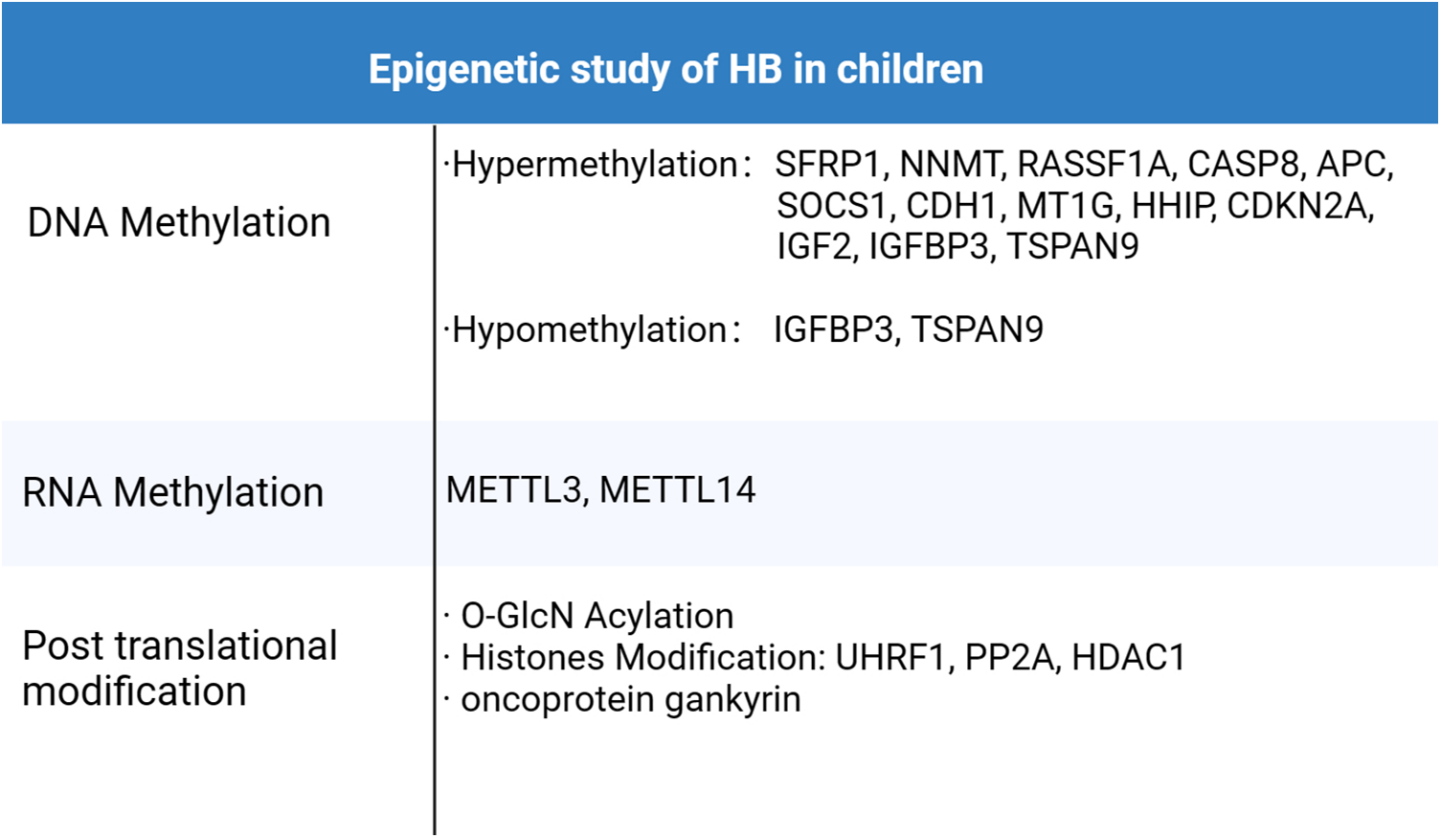
Epigenetic study of HB in children.

In future studies, it is essential to investigate whether other epigenetic processes contribute to the development of hepatoblastoma. Besides the epigenetic methods previously mentioned, there are additional processes like acetylation, phosphorylation, ubiquitination, sumoylation, and ADP ribosylation (30). We anticipate that further exploration will unveil more insights into the intricate relationship between hepatoblastoma and epigenetics. The epigenetic studies pertaining to HB discovered to date were summarized in Figure 3.

### 2.3 Transcriptomics studies of hepatoblastoma

In recent years, transcriptomics studies have yielded a wealth of valuable information regarding hepatoblastoma tumor specimens. Transcriptomics, as a field of study, is dedicated to capturing both coding and non-coding RNA and quantifying gene expression heterogeneity across cells, tissues, organs and even entire organisms. This approach is crucial because it forms the foundation for exploring regulatory pathways and genetic networks that govern both qualitative and quantitative phenotypes, with significant implications for human medicine.

Over the past decade, innovative technologies such as single-cell RNA-seq (scRNAseq), transwell analysis and spatial transcriptomics (ST) have been integrated into transcriptomics studies, leading to remarkable achievements. Recent transcriptome analyses have unveiled that the genes GATA4, DPEP1 and DRAM1 exhibit high expression levels in hepatoblastoma tumor samples. Moreover, studies have demonstrated that silencing the genes significantly inhibits the proliferation, migration or invasion processes of hepatoblastoma cells (54–56). Furthermore, another investigation has revealed that the expression of NTCP (SLC10A1) is downregulated in hepatoblastoma cells and tissues. Through experiments involving the transfection of hepatoblastoma cells with NTCP and subsequent cell proliferation assessments, it has been demonstrated that NTCP overexpression can induce apoptosis in hepatoblastoma cells, providing valuable insights into potential therapeutic targets (57).

In the realm of non-coding RNAs within the transcriptome, current research emphasizes three prominent categories: Long non-coding RNAs (lncRNAs), MicroRNAs (miRNA) and Circular RNAs (circRNAs). These RNA species play pivotal roles in the development of hepatoblastoma.

Long non-coding RNAs (lncRNAs) are non-coding transcripts exceeding 200 nucleotides in length. They exhibit tissue-specific expression patterns and serve as crucial regulatory factors in cellular physiology and pathology. Additionally, they have garnered attention as diagnostic and prognostic markers in various cancers (58). Numerous studies have underscored the unique contributions of multiple lncRNAs to hepatoblastoma development. A genome-wide analysis of lncRNA and mRNA expression identified 2,736 differentially expressed lncRNAs in hepatoblastoma tissues. Among these, 1,757 were upregulated, while 979 were downregulated (59). Notably, CRNDE expression was significantly elevated in hepatoblastoma samples and cell lines. Animal experiments revealed that CRNDE gene knockout markedly inhibited HB tumor growth and angiogenesis (60). Further investigation unveiled that CRNDE regulates hepatoblastoma angiogenesis by targeting the MiR-203/VEGFA axis (61). Another study demonstrated that PVT1 exhibited significantly higher expression in 43 human hepatoblastoma tissues compared to adjacent non-tumor tissues. PVT1 was found to promote hepatoblastoma cell proliferation *in vitro* and *in vivo*. Mechanistically, PVT1 accelerated the cell cycle progression of HB cells by activating STAT3, and inhibiting STAT3 effectively counteracted PVT1's effects on hepatoblastoma cell cycle progression and proliferation (62). Similarly, research has identified that lncRNAs TUG1, HOXA-AS2, OIP5-AS1, ZFAS1, SNHG9, NBR2 and MIR205HG are significantly upregulated in HB cells and enhance cell proliferation, migration or invasion in hepatoblastoma (63–69). Conversely, Gas5 was downregulated in HB cells, inducing cell apoptosis and inhibiting tumor growth through the activation of the CHOP-dependent endoplasmic reticulum stress pathway in human hepatoblastoma HepG2 cells (70).

MicroRNAs (miRNAs), small noncoding RNAs measuring approximately 22 nucleotides in length, play a pivotal role in post-transcriptional mRNA regulation. MiRNAs possess the capability to target a majority of mRNAs, rendering miRNA-mediated gene expression control a critical element in the cellular response to environmental stresses, such as hypoxia, starvation, oxidative stress, and DNA damage. Consequently, miRNAs have been implicated in human diseases, including cancer. An increasing body of research has identified numerous miRNAs that function as either oncogenes or tumor suppressors. Dysregulation in miRNA expression is closely linked to the initiation, progression, and metastasis of cancer. A comprehensive genomic analysis of hepatoblastoma tumors has unveiled 33 upregulated hub miRNAs; and 12 downregulated hub miRNAs, underscoring the significant role of miRNAs in hepatoblastoma formation (71). Ongoing research has demonstrated that mir-624-5p can markedly inhibit HB tumor growth by suppressing the transcriptional activity of Wnt pathway oncogenes (72). Additionally, another study has highlighted the involvement of miR-206 in T3-mediated regulation of lipid metabolism in HB cells (73). Furthermore, it has been observed that the overexpression of miR-206 negatively regulates the aggressive biological behaviors of HB cells, and this effect is partially reversed by KLB overexpression (74).

Circular RNA (circRNA) represents a novel class of endogenous RNAs found across various organisms and has been implicated in the development of various diseases, including neurological disorders, cancer, and prion-related diseases (75). In the context of hepatoblastoma, a study has identified 869 differentially expressed circRNAs between hepatoblastoma and adjacent normal liver samples. Among these, 421 were upregulated, while 448 were downregulated. Notably, the upregulated circRNA circ_0015756 was found to play a critical role in hepatoblastoma by acting as a sponge for miR-1250-3p, thereby regulating hepatoblastoma cell viability, proliferation, and invasion *in vitro* when silenced (76). Furthermore, other studies have highlighted the significance of upregulated circRNAs, such as circ_0000594, circ_0043800 and CDR1AS, in promoting the proliferation, invasion, and migration of HB cells. Silencing these circRNAs has been show to significantly inhibit the growth and migration of hepatoblastoma cells (77–79). Transcriptomics primarily explores potential factors contributing to hepatoblastoma at the RNA level. This section introduces the relationship between RNA and proteins related to HB, highlighting three categories of RNAs: lncRNA, miRNA and circRNA.

**Figure 4:**
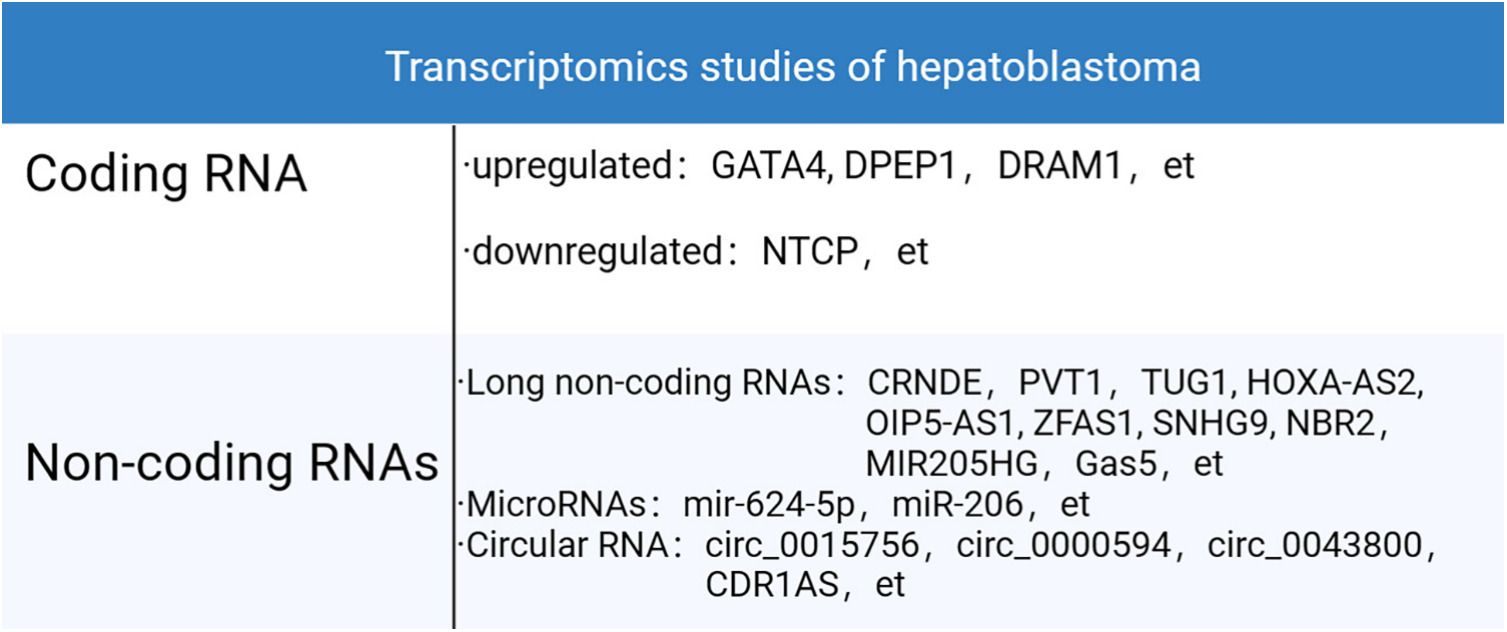
Transcriptomics studies of HB.

While current research in this area may appear somewhat fragmented, the maturation of technologies such as GO analysis, pathway analysis, miRNA response element analysis, and Transwell assays is gradually forming a comprehensive biological information network related to hepatoblastoma. In the future, it is anticipated that the issue of scattered information will be effectively addressed, leading to the development of more comprehensive biomacromolecule-based risk stratification, prognostic stratification, and treatment protocols for hepatoblastoma. The transcriptomics studies pertaining to HB discovered to date were summarized in Figure 4.

### 2.4 Molecular subtypes and clinical prognosis of hepatoblastoma

Children's Hepatic International Collaboration (CHIC) is renowned for developing the well-known PRETEXT risk stratification, utilizing factors such as the age of onset, tumor invasion extent, AFP level and metastatic status, etc. (80). Establishing molecular subtypes of tumor also holds immense importance in predicting tumor prognosis and guiding treatment strategies. In earlier microarray studies, hepatoblastoma was classified into two subclasses, C1 and C2, Using a 16-gene signature (50). Within this classification, C2 tumors were identified as a poor prognosis, while C1 tumors predominantly consisted of well-differentiated fetal histologies. In C1 tumors, β-catenin was primarily localized in the membrane and cytoplasm. Conversely, C2 tumors exhibited a higher proliferation rate, an enrichment of cell cycle-related genes, a prevalence of MYC target genes, frequent loss of membrane anchorage and pronounced nuclear aggregation. The aggressiveness of these tumors was associated with a liver stem-like phenotype and upregulated MYC expression. In a subsequent study, RNA sequencing of 25 tumors and matched normal liver samples from patients led to the identification of two subtypes: C2A and C2B. The C2B subtype exhibited increased expression of markers associated with epithelial-mesenchymal transition, including vimentin. On the other hand, the highly proliferating C2A subtype was characterized by upregulation of the topoisomerase 2-alpha gene and activation of the FA pathway activation (81). Combining clinical factors with the 16-gene signature, another study proposed a novel stratification system that facilitates risk-adapted management of HB patients (82). Significantly, this new system is currently in use to define risk stratification in the Paediatric Hepatic International Tumour Trial (PHITT).

**Figure 5:**
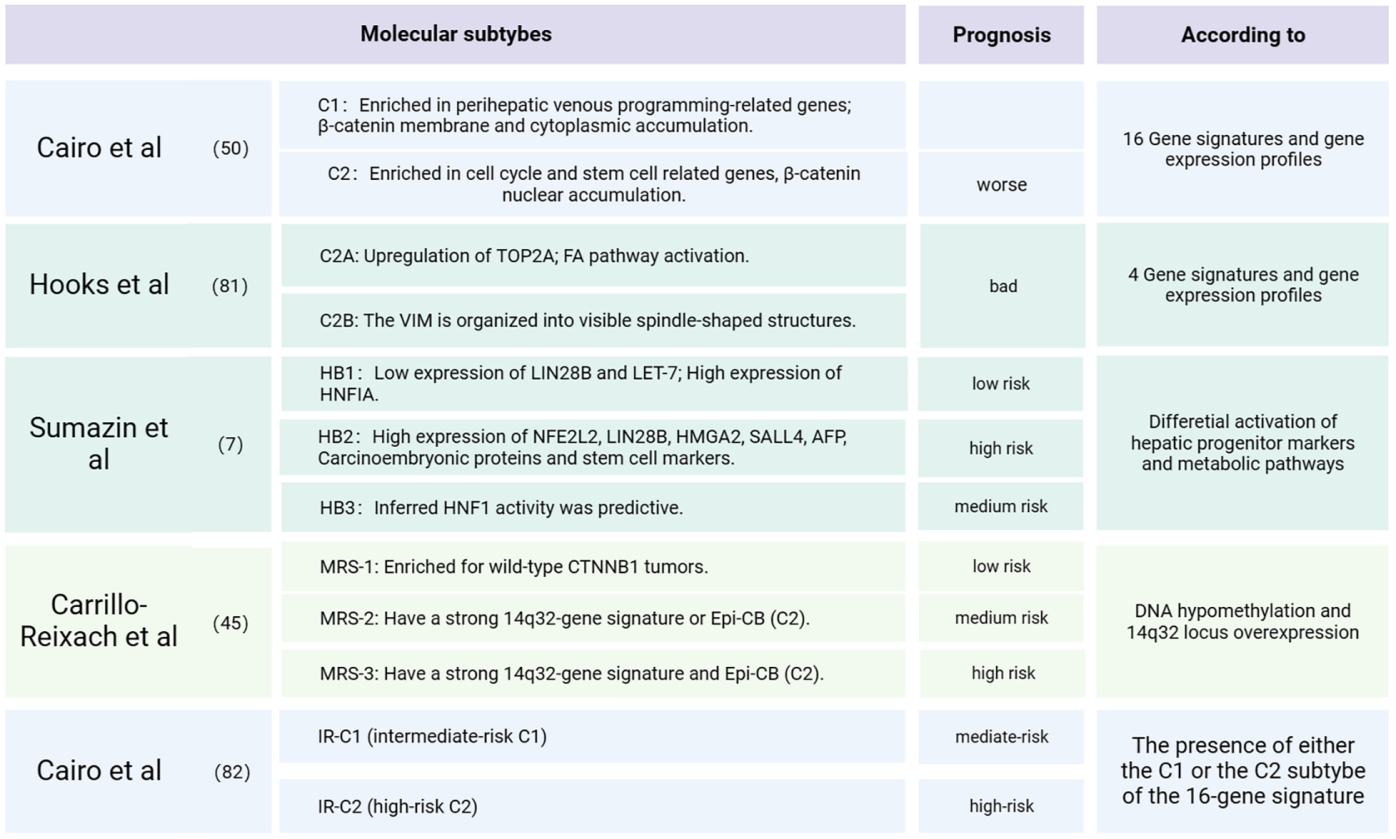
Molecular subtypes and clinical prognosis of HB.

In the quest to identify validated prognostic or therapeutic biomarkers for HB patients, an analysis of 88 clinically annotated HB cases has unveiled three distinct molecular subtypes that provide valuable risk stratification. These subtypes are characterized by differential expression of hepatic stem/progenitor markers (LIN28B, SALL4, AFP), hepatobiliary markers (HNF, NOTCH1), metabolic factors (NFE2L2), and cancer-related pathways (P53, TERT). These subtypes have been denoted as HB1 (low risk), HB2 (high risk), and HB3 (intermediate risk) (7). HB1 tumors are characterized by low expression of LIN-28 homolog B and lethal-7, along with high activity of hepatic nuclear factor 1 alpha. In contrast, HB2 tumors exhibit high expression of NFE2L2, LIN28B, HMGA2, SALL4, AFP, carcinoembryonic antigen, and stem cell markers. Interestingly, these three molecular subgroups exhibit some overlap with previously defined subgroups (50), with C2 tumors falling within the HB2 subgroup and C1 tumors falling within the HB1 and HB3 groups. Similarly, recent research has identified three distinct tumor subtypes: “proliferative”, “mesenchymal” and “hepatocyte”. The “proliferative” HB subtype predominantly expresses genes related to cell cycle regulation (CCNB2, E2F1, MKI67, MYCN) and AFP, indicating its aggressive nature characterized by robust cell proliferation. This subtype also shares a high expression of canonical Wnt-targeted genes (LGR5, TBX3, BMP4, ASCL2, RNF43, DKK1, SP5, NOTUM and GPC3), consistent with a high frequency of CTNNB1 mutations or deletions. The “mesenchymal” subtype is characterized by the presence of T cell receptors, matrix metalloproteinases (MMPs), and granzymes (GZMs), suggesting intratumoral infiltration of cytotoxic immune cells (CTLs, NKT and monocytes) along with extracellular matrix degradation. The “hepatocyte” subtype likely expresses genes associated with the metabolic functions of mature hepatocytes such as Cytochromes P450 (CYPs), UDP glucuronosyltransferases (UGTs) and metallothionein family genes (MTs) (10). The “proliferative” subtype exhibits similarities with the C2 subtype (50) of HB. Furthermore, recent findings have revealed significant plasticity among “hepatocytic”, “liver progenitor”, and “mesenchymal” molecular subgroups of hepatoblastoma. The “hepatocytic” subgroup contains more differentiated fetal components, while the “liver progenitor” subgroup is characterized by highly proliferative embryonal components. The “mesenchymal” subgroup includes tumors with mixed epithelial and mesenchymal features (15). These insights into molecular subtypes contribute to a deeper understanding of hepatoblastoma heterogeneity and hold promise for tailored treatment approaches. Figure 5 summarized the molecular-biological risk stratification of HB as detailed above.

## 3 Discussion

Over the past few decades, our comprehension of childhood hepatoblastoma has transitioned from clinical observations to the molecular level. The development and application of biotechnology have shed light on the pathogenesis of hepatoblastoma in children, with biological research accelerating this progress. As mentioned earlier, the cure rates for intermediate and high-risk patients remain less satisfactory. Furthermore, the chemotherapy drugs currently in use can impose a significant physical burden on children. Recent research has highlighted a concerning association between cisplatin and hearing loss, affecting over 50% of children with hepatoblastoma (83). Consequently, there is a pressing need to explore ways to mitigate the side effects of these chemotherapy drugs. According to a report from the International Childhood Liver Tumors Strategy Group (SIOPEL), out of 109 patients randomized to receive either cisplatin alone or cisplatin in combination with sodium thiosulfate, 63% of those who received cisplatin alone developed grade 1 or higher hearing loss, compared to only 33% of patients who received sodium thiosulfate in conjunction with cisplatin (84). Additionally, it is imperative to harness the advancements in molecular biology research pertaining to hepatoblastoma and drive the development of new targeted treatments. As mentioned earlier, Glypican-3 (GPC3), a canonical Wnt-targeted gene specifically expressed in hepatoblastoma (10), offers a promising avenue for targeted therapy. There are ongoing efforts worldwide to develop CAR-T therapy for solid tumors, with therapies based on GPC3 antibody genes (GPC3-CAR) already in development (85). Notably, Japanese researchers formulated a CPG3 vaccine and conducted clinical trials. The reports indicated that all five HB patients remained recurrence-free for more than four years (86).

Additionally, Substance P (SP), acting through the NK-1R receptor, functions as a universal mitogen in hepatoblastoma and regulates tumor growth (87). A recent study has identified Aprepitant, one of the NK-1R antagonists, as an efficient inhibitor of hepatoblastoma tumor growth by decreasing β-catenin levels and downregulating the Wnt target genes AXIN2 and LGR5 (88). Similar targeted drugs are continuously being developed and incorporated into clinical trials, fostering optimism that safer and more effective treatments for hepatoblastoma will emerge in the future.

## 4 Conclusions

To provide a comprehensive summary of the molecular mechanisms associated with hepatoblastoma occurrence, this review examines three key aspects: genomics, epigenetics, and transcriptomics. Within the realm of genomics, gene mutations, particularly those occurring in the CTNNB1 gene, have been identified as the primary drivers of hepatoblastoma. Additionally, chromosomal imbalances and single nucleotide polymorphisms also contribute to its development. In the domain of epigenetics, we focus on elucidating the roles of DNA methylation, RNA methylation, and protein modifications in hepatoblastoma formation. While the previous emphasis in transcriptomics research has been on coding RNA, investigations have highlighted the significant involvement of non-coding RNA in hepatoblastoma pathogenesis. This review provides a comprehensive overview of three categories of non-coding RNAs: lncRNAs, miRNAs, and circRNAs, which are currently at the forefront of research. Furthermore, this review summarizes the molecular subtyping of past hepatoblastoma cases. As clinical data continue to improve, prognosis analyses grounded in hepatoblastoma molecules markers will gain enhanced reliability. Finally, we address the present challenges associated with treating high-risk hepatoblastoma types and emphasize the imperative need to investigate the molecular biology mechanisms underpinning this condition. These molecular biology studies hold promise for the development of safer and more effective treatment methods in the future.
